# What type of client do you need? The brand value co-creation in the banking sector

**DOI:** 10.3389/fpsyg.2022.988985

**Published:** 2022-09-06

**Authors:** Nathalie Peña-García, Mauricio Losada-Otálora, Jorge Juliao-Rossi

**Affiliations:** ^1^Department of Research, Colegio de Estudios Superiores de Administración (CESA), Bogotá, Colombia; ^2^Departamento de Administración, Facultad de Ciencias Económicas y Administrativas, Pontificia Universidad Javeriana, Bogotá, Colombia; ^3^Facultad de Economía, Empresa y Desarrollo Sostenible-FEEDS, Universidad de La Salle, Bogotá, Colombia

**Keywords:** latent profile analysis (LPA), customer profiles, cross-cultural study, idiosyncrasy, brand value, co-creation, unobserved heterogeneity

## Abstract

Service-dominant logic established that for the success of service industries, it is vital to acknowledge the customer as an active agent in the commercial ecosystem. To carry it out, the consumer must participate in value creation. The resource integration theory exposes the importance of recognizing the customer as an agent capable of improving the company’s competitive advantage. It is only necessary for the participants to perceive benefits to make their resources available and integrate them into the co-creation process. This study aims to find the key customer-based factors that influence the brand value co-creation (VCC) process in the banking sector, analyzing the dynamics in different customers across national cultures and idiosyncrasies. In this paper, we analyze the potential heterogeneous idiosyncrasy of customers and how it leads to becoming more engaged in the co-creation process. Quantitative research was performed in five countries, obtaining a total of 2,029 valid questionnaires where latent profile analyses and ANOVAs were performed to identify and describe the latent profiles (LPA) of consumer co-creators of brand value. Afterward, a PLS-SEM was performed to test the research model in each segment. The results show four different profiles of customer co-creators of brand value, from non-co-creators (detractors), skeptical and neutral, to customers committed to co-creating brand value with their banks. The results indicate that detractor customers lack the motivations and resources to carry out co-creation behaviors. On the other hand, creativity and connectedness were crucial for customers co-creators of value. To the authors’ understanding, no studies have used latent segmentation to find the profiles of customer co-creators of brand value.

## Introduction

When “going to the bank” is on your to-do list for the day, what is the first emotion you experience? Indeed, it is not the same as buying that jewel, dress, or cake you have been dreaming of. For customers, doing errands in banks is not satisfactory; it is a fact. Banks are necessary but not close to the people, making it challenging to build a long-term relationships. Moreover, with the development of technology implemented in financial services and innovation in easily replicable processes, the banking market is at a high level of competition where customers have significant decision-making power. Therefore, banks must deliver more value to customers, allowing them to differentiate themselves from their competitors.

Furthermore, Larry Fink—CEO of Black Rock, the world’s largest investment fund—highlights the importance of Stakeholder Capitalism, which translates as the awakening of consciousness in capitalism: Companies must focus on the relationship with their stakeholders to create long-term value. That value must be co-created between producers and customers, employees and employers, partners and regulators (Mazzucato, 2022). In this work, we will approach the collaboration between customer and company to determine the motivational factors and the customer-owned resources necessary to promote customer brand value co-creation (VCC).

Financial services nowadays are framed in an ecosystem composed of fintech startups (such as payment technologies, loans, and crowdfunding, transaction and payment terminals, personal finance management), technology developers (such as big data analytics, digital currency, and cryptocurrency, and social networks, developers), government, financial customers, and traditional financial institutions (Lee and Shin, 2018). Moreover, in developed economies such as the United States, a conscious shift toward stakeholder capitalism is taking place (Fink, 2022). Hence, decisions are based on what benefits the bank’s stakeholders to provide long-term value to investors, with particular emphasis on strategies focused on customers to generate value in its user base (Casper Ferm and Thaichon, 2021).

For banks, gaining customer loyalty has been challenging due to the high exposure to electronic media, which keeps customers informed about the competition’s offers in real-time (Alam et al., 2021). However, these same electronic channels can allow customers from emerging economies to access banking services where market penetration is low (Hassan and Wood, 2020) wood and can also help for VCC processes, given their interactive nature. As a result, the bank customer is immersed in this digital ecosystem that, until a few years ago, was responsible for delivering information, which customers analyzed to make purchasing decisions. Now, that client is expected to become an active agent in the ecosystem, as it is based on the service dominant logic (SDL).

The SDL is an alternative and challenging view of the traditional neoclassical economics view of goods-dominant (GD logic), which considers firms as producers of goods/services capable of creating value (Cambra-Fierro et al., 2017). According to Vargo et al. (2008), value is created by the firm and distributed in the market. Therefore, the roles of producers and consumers are not the same. In contrast, the SDL considers producers and consumers equal, and value is co-created simultaneously and mutually. SDL was initially proposed by Vargo and Lusch (2004), and it has been studied in the last decade as a specialized area of service marketing (Vargo and Lusch, 2017). Authors like Prahalad and Ramaswamy (2004) have encouraged the study of VCC as the SDL key since it allows firms to offer more and better value to the market (Cambra-Fierro et al., 2017). Considering customer participation in the production, distribution, and development processes of new products and services can improve the profitability of companies (Moghadamzadeh et al., 2020). According to Al-Kumaim et al. (2021), managers must continuously learn to acquire new tools that motivate their clients to co-create value because, as Karpen et al. (2012) affirm, the implementation of these processes becomes a competitive advantage vital to companies.

According to Bruce et al. (2019), one of the priorities for consumer behavior researchers and practitioners is to “understand how multiple actors combine to create value” (p. 173). According to resource integration theory (Kleinaltenkamp et al., 2012), for a customer or any actor to become an active agent in co-creation processes, they must have specific resources on brands, like knowledge and skills, and the willingness to deliver them to the company. In that sense, this work follows the approach of Merz et al. (2018). They affirm that brand VCC is possible solely when customers show high levels of customer-owned resources and motivations like trust, passion, and commitment.

This work seeks to contribute to the knowledge of the brand VCC process, specifically in the banking sector, which experiences strong emotions from its users and can be challenging to maintain long-term relationships with them, given its sensitivity to price (rates). Understanding that customers can act differently because of their previous experiences, the construction of their beliefs, personal values, and ultimately, their idiosyncrasies, we present a latent-segmentation study to find the unobserved heterogeneity of customers based on their idiosyncrasy and national culture.

In this sense, the research questions that will guide this work are:

*RQ1. What customer-owned resources drive them to co-create brand value with their banks?*

*RQ2. What customer motivations drive them to co-create brand value with their banks?*

The study is structured as follows: first, we present a literature review of the main topics for this research, namely, brand VCC, national culture, and idiosyncrasy. Second, the research method is presented. With data obtained in five countries, an analysis of latent profiles (LPA) is made based on the level of co-creation of the surveyed consumers and their idiosyncrasy, getting four differentiated profiles that we named (1) detractors customers, (2) skeptical customers, (3) neutral customers and, (4) customers co-creators of brand value. Third, in the discussion of results, the four profiles obtained are analyzed to expand knowledge about brand VCC. We found that the ultimate resource for brand VCC is creativity, while brand knowledge and skills are not determinants of behavior. Finally, the research’s main conclusions, implications, and limitations are presented.

## Theoretical background and hypotheses

To carry out the literature review, a systematic process was followed based on the research questions and the aim of the study. Thus, variables to be considered to solve the questions and achieve the goal of the investigation were determined. As the study of brand VCC might be broad, we focus on studies carried out in the banking sector since its nature makes it significantly different from studies carried out in other contexts. Subsequently, we review the papers that work on the research topic and are published in high-impact journals (Q1 and Q2). The studies found in the banking sector are valuable but scarce, so we complement the literature review with studies in other sectors to understand the differences that underlie these contexts and thus help fill the gap in this type of study.

Thus, a review of the literature on brand VCC is presented to determine the customers’ intrinsic factors influencing this behavior, as well as the idiosyncrasy and national culture, with which it is planned to carry out the analysis of unobserved heterogeneity.

### Brand value co-creation

Relational marketing establishes perceived value as the key to maintaining long-term customer relationships (Aaker, 1996). The SDL transcends the role of the customer as an active agent co-creator of value (Payne et al., 2008). The first perspective assumes the company must study the consumer and, according to the results, generating value keeps the customer interested in the business relationship, making them taxable. The SDL understands the customer as an active and involved subject in the value creation and exchange process. In that order, the value is not exchanged between actors but co-created (Pohlmann and Kaartemo, 2017).

Vargo and Lusch (2016) describe VCC as how value is created from the interaction between agents participating in an exchange process. Nysveen and Pedersen (2014) defined co-creation as a mutual comprehension process wherein one hand, companies develop what consumers expect to get. On the other hand, consumers select or modify services according to their needs.

The study of brand value has been an extension of these theories. Merz et al. (2018, p. 79) describe brand value “as the value that is solely attributable to a brand.” As the formal study about perceived value, the brand value may be evaluated just for the receivers, being subjective, personal and may differ from one customer to another. Brand value is the perceived use value resulting from the customer’s experience with the product or service, which is attributed solely to the brand (Merz et al., 2018). From Merhabi et al. (2021) perspective, brand VCC refers to customer involvement “in business-related activities, such as promoting, advocating, collaborating, and sharing knowledge with their companies” (p. 3). Despite the competitive advantage that adopting co-creation brand value processes may give any company, especially the service companies, there is scarce research on the subject.

Previous research has emphasized the need for service companies to pay more attention to the resources that customers can contribute to companies and offer suitable scenarios for joint VCC (Al-Kumaim et al., 2021). Likewise, the challenge companies face, especially service providers, to attract and motivate customers to participate in VCC processes has been pointed out (Monavvarifard et al., 2019). However, it is not placed greater emphasis on the capacities that the client must have to participate in such processes. According to Merz et al. (2018), the process of co-creation of value in clients arises from two perspectives: customer-owned resources and motivational factors.

Laid on SDL theory from Vargo and Lusch (2004) and their study on co-creation with stakeholders (Vargo et al., 2008), Kleinaltenkamp et al. (2012) proposed the resource integrators theory, which recognizes the role of the different actors who participate in company networks. According to Kleinaltenkamp et al. (2012) the resource integrators are all the actors (individuals or organizations) with an agency. That means any actor with an operant resource willing to offer and integrate it with another actor. The willingness to participate in resource integration generally relay on the perceived benefits. Customers must perceive that the bigger and stronger the company, more benefits for the customers. On the other hand, companies must understand that even when opening to external opinions and decisions may be difficult, sometimes expensive, and to create conflicts in every level, the integration of their stakeholders, specially customers, is going to give them the competitive advantage to remain active in the market.

In their work, Merz et al. (2018) focus on creating a scale that helps measure customer value in brand VCC considering that the literature has traditionally exposed two ways to understand the intention of customers’ VCC: their abilities and their willingness. Abilities are also known as customer-owned resources, which refer to those resources that customers have and companies want, such as knowledge and brand skills. His study is based initially on the customer engagement literature and refers to the customer’s willingness to participate in brand-building activities. However, it also receives support in the theory of integrated resources, which indicates that clients must understand and assume a role within the co-creation process (Vargo and Lusch, 2004). By identifying themselves as active users, they may take companies’ resources and use them to co-create.

The willingness addresses customers’ motivations to deliver these resources to companies. In other words, for a client to enter into a brand VCC process, they need the necessary resources to do so and be motivated. Motivations have been extensively studied in brand relationship quality and are proposed in Merz as passion, commitment, and trust.

In the light of the studied literature, customer-owned resources are:

• Brand knowledge: refers to the whole set of experiences the consumer has about the brand.
• Brand skills: refers to customer perception of the capabilities of the company.
• Brand creativity: represents the “production, conceptualization, or development of novel and useful ideas, processes, or solutions to problems” (Kozinets et al., 2008).
• Brand connectedness: refers to the degree to which a consumer is in capacity and seeks to be in a relationship with other users or customers of the brand.

On the other hand, dimensions of customer motivation, according to Merz et al. (2018) are:

• Passion: refers to positive and strong feelings from a customer toward a brand, such as love or admiration.
• Trust: refers to the consumer’s belief about the brand’s benevolence, its capability in fulfilling its promises, and in general, the confidence in the brand
• Commitment: The degree to which the consumer is committed to helping the brand be a success

In the Merz study, the scale is built to measure the customer value in the brand VCC process, understanding it as a multidimensional and formative construct. In this work, we seek to study the intention of brand VCC from the dimensions proposed by Merz since it is established that the dimensions have been proven as the determinant factors for brand VCC. Thus, in this study, the following hypotheses are proposed.

H1. Brand knowledge has a positive and significant effect on value co-creation.

H2. Brand skills have a positive and significant effect on value co-creation.

H3. Brand creativity has a positive and significant effect on value co-creation.

H4. Brand connectedness has a positive and significant effect on value co-creation.

H5. Passion has a positive and significant effect on value co-creation.

H6. Trust has a positive and significant effect on value co-creation.

H7. Commitment has a positive and significant effect on value co-creation.

**FIGURE 1:**
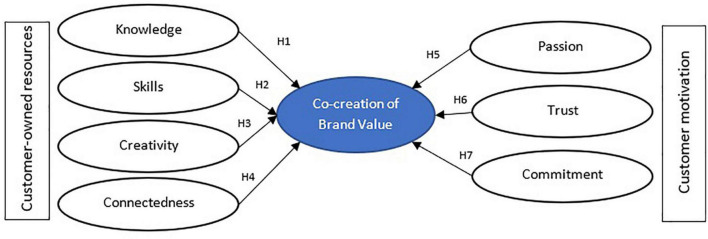
Research model.

Research model is presented in Figure 1.

### Unobserved heterogeneity

In the study of consumer behavior, the need to segment customers according to their heterogeneous behavior patterns has been continuously highlighted (Fuentes-Blasco et al., 2014). Unobserved heterogeneity can cause theoretical mismatch and oversimplify the underlying complexity of individual differences (Sarstedt et al., 2022). This is why in this research, we use idiosyncrasy to detect unobserved heterogeneity and generate customer segments. Likewise, we take the national culture to understand the differences between the sub-groups that make up the sample.

#### Idiosyncrasy

According to Gorgoglione and Panniello (2018), the depth research of customer experience may allow identifying idiosyncratic perceptions to study groups of consumers. Although national culture should define consumer behavior, personal factors may shape how a consumer perceives the experience, and thus, the outcomes of the experience may vary among individuals. Idiosyncrasy has not been a popular variable in marketing research; previous research has only used it as an argument to explain personal differences between groups of individuals (Moschieri and Campa, 2014). Although idiosyncrasy is fundamentally a personal factor, Hollander (1958) proposes the existence of a “group idiosyncrasy” that develops from the subjective elements of the individuals. Their expectations about what is expected to be an appropriate behavior or attitude to continue belonging to the group.

Idiosyncrasy may be defined as the beliefs, attitudes, and behavior of an individual or group regarding another individual or group. In this research, we define idiosyncrasy as the beliefs and attitudes of a consumer regarding the bank services that may lead to positive and negative behaviors. Fourie (2012) associated idiosyncrasy with uniqueness, peculiarity, unconventionality, and individuality. Each person shows their idiosyncrasy through technology. Therefore, it suggests more studies that relate ICT and idiosyncrasy since how people use their technologies will give more information about this topic that has been scarcely studied in the literature on consumer behavior. Therefore, the information could be analyzed to promote innovation, productivity, and efficacy. Thus, idiosyncrasy should be deeply studied so the banks can establish and maintain long-term business relationships with their customers.

Gorgoglione and Panniello (2018) found that idiosyncrasy may lead to establishing clusters of customers according to their perceived experience in bank services. Therefore, in this investigation, idiosyncrasy as a critical element of the experience perceived in the VCC will be studied to generate the profiles of co-creative consumers.

#### National culture

Academia culture studies are more developed in countries like the United States and Spain. In contrast, studies about South Africa, India, and Japan are more recent and date back to 1980 (Burton, 2008). In Latin America, research can be found since the early twentieth century; however, the actual emergence of the discipline began in the 1970s (Hart and Young, 2014) since the history of Latin America differs totally from the history of the rest of the world. Latin America has had a significant influence from Europeans, Asians, and Africans. After the conquest and independence of the countries, there was no national culture but a combination of symbols, traditions, and customs from the conquering nations. Subsequently, conquered and liberated countries would take more than a century to acquire their own national culture.

Several studies have found that culture affects marketing, explicitly advertising, marketing strategies, and consumption habits (Haffar et al., 2016; Rehman, 2017; Robinson, 2019). In addition, the relevance of culture in consumer behavior research is based on the influence of culture on lifestyle, which in turn influences the communication and interaction of individuals with IT (Brandtzæg, 2010).

Culture has proved to be a problematic term for academics to define, and social scientists accept no universal definition. Its complexity has allowed several authors to provide concepts to literature from different perspectives (Burton, 2008). On the other hand, Williams (2014, p. 87) declares: “culture is one of the two or three most complicated words of the English language.” Social scientists have used the concept to refer to a set of parameters that a group shares and differentiates it from another significantly (Brodbeck et al., 2013). According to Steers et al. (2008), culture shares values, norms, and mutual behavior patterns. Despite culture being invisible, some characteristics and manifestations are indirectly recognized.

The word nation is deeply related to the native term, which refers to the birth of the human being in a fabric of relationships that settle in a geographical place (Williams, 2014). Hroch (1998, p. 79) defines it as “a large social group integrated not by one, but by a combination of various types of objective relationships (economic, political, linguistic, cultural, religious, geographical, historical) and their subjective reflection on the collective consciousness.”

The national culture as a concept is relatively new, and some reject the idea due to the growing flow of information that permeates the culture of small populations that make up a nation. However, the idea that each nation has a distinctive culture is widely accepted, and the use of national culture as a unit of geographic analysis is widespread in marketing (Burton, 2008). Besides, some authors claim that national culture can be a great tool to examine the generalization of theories in marketing and reveal their conditions according to the environment (Engelen and Brettel, 2011).

Following Kumar and Pansari (2016), national culture may influence consumer behavior, especially in service industries. Additionally, some authors consider that new technologies can make similar societies when different societies turn modern and become similar developments. However, the same technology development may increase differences because, first, it can change pre-existing value systems; second, societies face different ways of technological modernization (Hofstede, 2011).

In this research, the national culture will be a critical variable in the differentiation of VCC behavior in the banking industry of five nations. We found various models in the literature to measure the national culture, especially Hofstede, GLOBE, and Schwartz. Hofstede’s cultural dimensions (Hofstede, 1994, 2009, 2011) define culture as “the collective programming of the mind which distinguishes the members of one group from others,” the model consists of 63 items administered to more than 116,000 employees of IBM of 40 countries and presents a structure of four original dimensions: individualism vs. collectivism, power distance, masculinity vs. femininity, and uncertainty avoidance. Later on, based on the findings of the Chinese Value Survey, a fifth dimension was added to evaluate time orientation within a culture: long-term vs. short time orientation (Hofstede and Bond, 1988; Hofstede, 2011).

The Global leadership and Organizational Behavior Effectiveness, or GLOBE research program, was designed by Robert J. House in 1991. Volume I, dedicated to culture, leadership, and organizations, presented the dimension resulting from 17,300 intermediary managers from 951 local organizations in 62 societies worldwide. Later, in volume II, they studied 25 companies and completed the conclusions of volume I. The model identifies nine cultural dimensions (Tung and Verbeke, 2010; Carolina, 2019). Most of the nine dimensions are based on the Hofstede model.

Finally, the Schwartz model (1992) differs from other cross-cultural models by combining human societies’ values and cultural orientations. The study set up a list of 57 value elements for students and teachers in more than 70 countries (Guo et al., 2020). In this investigation, we decided to use the dimensions of Schwartz since its objective is to present a theory that could potentially be universal toward aspects of human values (Schwartz, 1994) also because inconsistency is found in Hofstede’s model and, consequently, the GLOBE model. For example, Brewer and Venaik (2012) warn that the national-level culture dimensions do not apply to organizations or individuals across nations. In other words, the model works only at the national level. But this is something that, as both Hofstede and GLOBE acknowledge in their research books and journal papers, several studies still apply the model to individuals.

Schwartz (1999) argues that values are answers to three fundamental needs faced by human beings and society: (1) biological needs, (2) the need for coordinated social interaction, and (3) the need for proper functioning and survival of the group. Schwartz (1994) proposes that there are 10 types of motivational values categorized into self-transcendence (universalism and benevolence), self-enhancement (achievement and power), conservation (tradition, conformity, and security), and openness to change (stimulation and self-direction) (Ahmad et al., 2020). The 10 types of motivational values are presented in Table 1.

**TABLE 1:** Ten types of motivational values—Schwartz.

Definition	Examples	Source
Power: Social status and prestige, control or control over people or resources	Authority of social power, wealth	Interaction, group
Achievement: Personal success demonstrating competence according to social standards	Successful, capable, ambitious	Interaction, group
Hedonism: Pleasure and sensual gratification for itself	Pleasure, enjoy life	organism
Stimulation: Emotion, novelty, and challenges in life	A varied, daring, exciting life	organism
Self-direction: Independent thinking and action - choose, create, explore	Creativity, curiosity, freedom	Organism, interaction
Universalism: Understanding, appreciation, tolerance, and protection for the wellbeing of all people and nature	Tolerance, social justice, equity	Group, organism
Benevolence: Preservation and improvement of the wellbeing of people with whom frequent contact is maintained	Social, honest, indulgent	Organism, interaction, group
Tradition: Respect, commitment, and acceptance of customs and ideas that provide traditional culture or religion	Humble, devout, accepting position in life	Group
Conformity: Containment of actions, inclinations, and impulses that may anger or harm others and violate the social expectations of the norms	Polite, obedient, honor parents and elders	Interaction, group
Security: Harmony, security, and stability of society, relationships, and self	National security, social order, cleaning	Organism, interaction, group

Organism, universal needs of individuals as biological organisms; Interaction, universal requirements for coordinated social interaction; Group, universal requirements for the proper functioning and survival of the groups.

Schwartz (1994, p. 22).

These values can be compared to the dimensions proposed by Hofstede (2009), such as individualism and power distance. Therefore, the expectation of Schwartz (1994) is that the theory developed from his study can be applied to cross-cultural researchers to choose the samples strategically, based on the types of values.

## Research methodology and data collection

### Sample

Data was collected by a market research company in five countries using a structured questionnaire survey to find the factors influencing VCC behavior. Countries were selected based on several criteria. First, we wanted to compare Western countries with developed and developing economies, so no vast cultural differences became impossible to compare. In this sense, countries of America and Western Europe were considered. Then we reviewed the FinTech report from Statista.com (FinTech, n.d.), which shows the growth in the value of transactions carried out through web pages, apps, and other channels offered by banks from the leading countries since 2017 and projected until 2025. Finally, we found that the countries that met the geographic location requirement were: Argentina, Brazil, Canada, Chile, Colombia, Denmark, Dominican Republic, France, Finland, Greece, Italy, Mexico, Netherlands, Peru, Portugal, Spain, United Kingdom, and the United States. With this list of countries, we take a representative country for North America (United States) and Europe (Spain), and three from Latin America, an area primarily forgotten by research on marketing and behavior but of great interest for its growth. Thus, we choose the three most significant countries in Latin America according to their GDP: Brazil, Mexico, and Argentina (Latin America and Caribbean: GDP by country 2020, n.d.).

From Argentina and Spain, 403 valid questionnaires were obtained individually, from Mexico 401 and Brazil, and United States 421, obtaining 2029 valid observations for the study. All the ethical guidelines for data collection, informed consent, and appropriate disclaimers were reviewed and approved by the ethics committee of CESA. To participate in the survey, the respondents had to be active clients of a bank with at least one current product. In addition, they must have used two or more different channels of the bank to make their transactions in the last 60 days, be over 18 years old, and nationals or residents with more than 15 years of living in the country to avoid differences in the national culture of each subsample.

### Scales

The questionnaire was designed based on scales found in the literature that have shown in previous studies to have the reliability and validity necessary to be replicated. Thus, the scale to measure the antecedents of brand VCC (knowledge, skills, creativity, connectedness, passion, trustworthiness, and commitment) was adapted from Merz et al. (2018). To measure the brand VCC in bank customers, the study proposes a scale to measure the behaviors resulting from co-creation. This scale was created based on Payne et al. (2008) and Pinho et al. (2014). The scale was revised by experts and tested on a sample of 400 banking customers before the study. The 34 items of the idiosyncrasy scale were adapted from Gorgoglione and Panniello (2018). Finally, Schwartz’s work was used to measure national culture (Schwartz, 2012). Items are presented in Table 2.

**TABLE 2:** Scales for measurement instrument.

Construct	Dimension	Item	Author
Antecedents of value co-creation	Knowledge	I am informed about what my bank has to offer	Merz et al., 2018
		I am knowledgeable about my bank	
		I am an expert in things related to my bank	
	Skills	I think analytically when I deal with my bank	
		I think logically when I deal with my bank	
		I think critically when I deal with my bank	
	Creativity	I become imaginative when I interact with my bank	
		I become creative when I interact with my bank	
		I become curious when I interact with my bank	
	Connectedness	I am networked with other customers of my bank	
		I am connected to other customers of my bank	
		I belong to one or more bank communities related with my bank	
		I socialize with other customers of my bank	
	Passion	I am addicted to my bank	
		I am a fan of my bank	
		I love my bank	
		I admire my bank	
	Trust	I trust my bank	
		My bank addresses my concerns honestly	
		I rely on my bank when I have a financial problem	
		I depend on my bank to satisfy my financial needs	
	Commitment	My goal is to make my bank a success	
		I am driven to make my bank a success	
		I am committed to making my bank a success	
		I am enthusiastic about making my bank a success	
Value co-creation		I normally read the publications made by my bank on its website or social media	Based on Pinho et al., 2014; Payne et al., 2018
		I publish original content about my bank through my social networks	
		I share information about my bank through my social networks	
		When I purchase a financial product in my bank, I can customize it according to my interests	
		When I want to buy a financial product or use any of my bank’s services, and I have any concerns, I use a customer service channel (chat, call, email, etc.)	
		I identify myself with the values of my bank	
Idiosyncrasy		I became a customer of my bank because it was recommended to me	Gorgoglione and Panniello, 2018
		All I care about is which bank gives me the best financial conditions (rates) for what I need	
		I would much rather deal with someone face to face than over the phone, especially in financial matters	
		It would be great if I could deal with one designated sales rep throughout my relationship with the bank	
		I do not choose it by the rate alone; there are other important factors, like time and effort, too	
		It is important to me that the company I am dealing with is “local”	
		I want to choose between different options to make certain I get the best offer	
		It is more important to get what I need than to shop around for a better rate	
		I have dealt with them before so getting what I needed was really easy	
		Yes, there are other banks, but I would rather stay with mine; it makes the process much easier	
		I do not care about a relationship with this company; I just want the best rate	
		I stay with my bank because I am not confident using another one	
		It is important to me that the company I am dealing with has a good reputation	
		It is important that I am kept informed of what is going on throughout my dealings with the bank	
		It is important that the bank is sincere and explains the investment product in detail, making it transparent to me	
		It is important that they keep me up-to-date and inform me about new options	
		It was important that the sales rep knew what I was going through and could relate to it	
		Dealing with different forms and different people is not really “customer-friendly”	
		I choose different banks for different products to spread the risk	
		I want to have a guaranteed capital, a guaranteed investment	
		The whole process was so easy, they took care of everything	
		The way the deal with me when things go wrong will decide if I stay with them	
		It was important that they guided me throughout the whole process	
		It is important that the people I am dealing with are good people; they listen, are polite and make me feel comfortable	
		I am already a customer; they know me and take good care of me, there is no need for me to go somewhere else	
		It is not just about the now; this bank will look after me for a long time	
		I will not do business with pushy sales people	
		It is important that the bank was flexible in dealing with me and looking out for my needs	
		I choose them because they give independent advice	
		I want to deal with a safe Company, because this is my money	
		I did not receive any guidance and as a result I will look for someone else in the future	
		I am confident in their expertise, they know what they are doing	
		It was important to me that the bank also took care of all the other products I needed	
		If the advisor changes company I will consider moving my accounts with him/her	
Cultural values	Power	Social power, authority, wealth	Schwartz, 2012
	Achievement	Success, capability, ambition, influence on people, and events	
	Hedonism	Gratification of desires, enjoyment in life, self-indulgence	
	Stimulation	Daring, a varied and challenging life, an exciting life	
	Self-direction	Creativity, freedom, curiosity, independence, choosing one’s own goals	
	Universalism	Broad-mindedness, beauty of nature and arts, social justice, a world at peace, equality, wisdom, unity with nature, environmental protection	
	Benevolence	Helpfulness, honesty, forgiveness, loyalty, responsibility	
	Tradition	Respect for tradition, humbleness, accepting one’s portion in life, devotion, modesty	
	Conformity	Obedience, honoring parents, and elders, self-discipline, politeness	
	Security	National security, family security, social order, cleanliness, reciprocation of favors	

Except for Cultural Values, all the scales were measured with 7-point Likert-type scales where 1 is “totally disagree,” and 7 is “totally agree.” Cultural values were measured with a 0–8 point scale, where 0 is “opposed to my principles,” 1, 2, and 3 are “not important,” 4, 5, and 6 “important,” and 7 and 8 “of supreme importance.”

### Methods

The research proposes an LPA analysis to find the unobserved heterogeneity. LPA lets us obtain profiles of bank customers co-creators of brand value according to their idiosyncrasy, which provides differentiated segments with unique characteristics. ANOVA analyses and cross tables are used to find the antecedents of co-creation for each segment and national culture differences. The research model will be validated with the segments established using a PLS Algorithm analysis. Finally, the model will be tested in each customer segment to understand the factors influencing VCC.

## Results

### Estimating the number of profiles

To determine the correct number of profiles, the authors used mPLUS 8.3 to calculate the optimal number of statistically significant segments different from each other. We started with two segments, and we increased until the significance of the segments was above 0.05. Finally, seven fit indices were applied to know the optimal solution using TECH11 and TECH14. Table 3 shows the results.

**TABLE 3:** Comparison of fit indices for determining the number of classes.

Segments	LL	AIC	BIC	ABIC	ENT	LMRT *(p)*	BLRT *(p)*
2	−126,944	254,094	254,672	254,345	0.951	0.000	0.000
3	−124,185	248,646	249,421	248,982	0.951	0.004	0.005

4	−121,611	243,569	244,540	243,991	0.952	0.000	0.000
5	−120,489	241,395	242,563	241,902	0.939	0.000	0.000
6	−119,471	239,428	240,793	240,021	0.928	0.001	0.001
7	−118,887	238,331	239,892	239,009	0.925	0.445	0.446

LL, log likelihood; AIC, Akaike information criteria; BIC, Bayesian information criteria; ABIC, Adjusted Bayesian information criteria; ENT, entropy; LMRT, Lo-Mendell-Rubin-adjusted-likelihood ratio test; BLRT, parametric bootstrap likelihood ratio test.

According to Gabriel et al. (2015), the best model should have LL, AIC, and BIC fit lower than the other solutions and entropy more significant compared to other solutions. Also, LMRT and BLRT must be significant (*p* < 0.05). The fits of the model presented in Table 1 indicate that even when solutions from 2 to 6 classes are valid (*p* < 0.05), the solution of 7 classes is not feasible because LMRT and BLRT fits are *p* > 0.05. On the other hand, solutions of 2, 3, 4, 5, and 6 classes are viable, and segments are statistically significant. Still, due to the classes’ size and the highest entropy score, the 4-segments solution is the most accurate for this work, with 87, 524, 609, and 808 observations, respectively.

### Mixture regression

Table 4 shows the mixture regression of VCC with the proposed variables related to this research. The table presents the mean, the significance of the relationship, the number of observations, the percentage of the segment size on the sample, the most relevant idiosyncratic perceptions in each segment, cultural values, and country of origin. With these variables, each segment will be characterized.

**TABLE 4:** Mixture regression for four classes.

	Mean	Sig.	Counts	Counts%	Idiosyncratic perceptions	Cultural values	Country %
Segment 1/Detractors	2.474	0.000	87	4.3	Rates: 2.2 Personal: 2.8 Time and effort: 2.2 Comparing: 2.4 Loyalty: 2.3 Brand reputation: 2.4 Information: 2.2 Security and trust: 2.2 Ease process: 2.3 Crisis management: 2.3 Attention: 2.3 Product portfolio: 2.4	POW = 2.5 ACH = 3.5 HED = 3.0 STI = 3.3 SELF.D = 3.8 UNIV = 3.5 BEN = 4.3 TRAD = 3.5 CONF = 3.5 SEG = 3.90	Arg. = 19.5
VCC/knowledge	3.6						Spain = 23.0
VCC/skills	4.3						Mex = 17.2
VCC/creativity	2.2						Bra = 23.0
VCC/connectedness	1.5						United States = 17.2
VCC/passion	1.6						
VCC/trustworthiness	2.4						
VCC/commitment	1.6						
Segment 2/Skepticals	3.264	0.000	524	25.8	Rate: 4.3 Personal: 4.3 Time and effort: 4.4 Comparing: 4.6 Loyalty: 4.0 Brand reputation: 4.6 Information: 4.5 Security and trust: 4.3 Ease process: 3.5 Crisis management: 4.3 Attention: 4.6 Product portfolio: 4.4	POW = 2.9 LOG = 4.5 HED = 4.3 STI = 4.6 SELF.D = 5.9 UNIV = 6.0 BEN = 6.7 TRAD = 4.1 CONF = 4.1 SEG = 6.2	Arg = 29.7
VCC/knowledge	4.3						Spain = 22.4
VCC/skills	4.7						Mex = 18.8
VCC/creativity	2.6						Bra = 21.7
VCC/connectedness	1.7						United States = 7.4
VCC/passion	2.1						
VCC/trustworthiness	3.2						
VCC/commitment	2.0						
Segment 3/Neutral	3.868	0.000	609	30.1	Rate: 4.3 Personal: 4.7 Time and effort: 4.6 Comparing: 5.6 Loyalty: 4.5 Brand reputation: 5.8 Information: 6.2 Security and trust: 5.3 Ease process: 4.3 Crisis management: 5.4 Attention: 6.3 Product portfolio: 5.2	POW = 3.3 LOG = 4.2 HED = 3.9 STI = 4.0 SELF.D = 4.7 UNIV = 4.5 BEN = 5.0 TRAD = 4.9 CONF = 5.1 SEG = 5.1	Arg = 18.0
VCC/knowledge	4.5						Spain = 21.3
VCC/skills	5.1						Mex = 12.9
VCC/creativity	3.3						Bra = 18.9
VCC/connectiveness	2.4						United States = 29.0
VCC/passion	3.0						
VCC/trustworthiness	3.9						
VCC/commitment	2.9						
Segment 4/Brand value co-creators	4.755	0.000	809	39.9	Rate: 5.5 Personal: 5.5 Time and effort: 5.9 Comparing: 6.2 Loyalty: 6.1 Brand reputation: 6.4 Information: 6.5 Security and trust: 6.1 Ease process: 5.9 Crisis management: 6.1 Attention: 6.5 Product portfolio: 6.2	POW = 3.9 ACH = 5.2 HED = 4.8 STI = 5.1 SELF.D = 6.2 UNIV = 6.1 BEN = 6.7 TRAD = 5.8 CONF = 5.9 SEG = 6.7	Arg = 15.0
VCC/knowledge	5.6						Spain = 16.8
VCC/skills	5.7						Mex = 25.8
VCC/creativity	4.4						Bra = 18.8
VCC/connectiveness	2.8						United States = 23.6
VCC/passion	4.2						
VCC/trustworthiness	5.3						
VCC/commitment	4.2						

Results show four different segments of consumers co-creators of brand value according to their willingness to co-create. The first segment, we have denominated non-creators of brand value or detractors customers. The second segment has been denominated skeptical customers, the third segment neutral customers, and the fourth segment is composed of the actual customers co-creators of brand value.

### Model validation

A PLS Algorithm analysis was performed with SmartPLS 3.0 software to validate the model. The model fit showed satisfactory values. The composite reliability of the model is verified with values between 0.870 and 0.977 for all the factors. Cronbach’s alpha values are between 0.813 and 0.96. Also, AVE values are between 0.573 and 0.915. The values can be consulted in Table 5, including Fornell-Larcker criteria, to confirm discriminant validity.

**TABLE 5:** Reliability and validity of the model.

	KNO	SKL	CNN	CRT	PAS	TRS	CMM	VCC	Cr. α	CR	AVE
KNO	0.864								0.831	0.899	0.747
SKL	0.562	0.878							0.852	0.910	0.772
CNN	0.312	0.191	0.903						0.925	0.947	0.816
CRT	0.498	0.390	0.584	0.930					0.921	0.950	0.864
PAS	0.474	0.271	0.553	0.606	0.895				0.916	0.941	0.801
TRS	0.544	0.313	0.396	0.530	0.753	0.843			0.863	0.907	0.711
CMM	0.458	0.275	0.581	0.661	0.763	0.717	0.956		0.969	0.977	0.915
VCC	0.507	0.367	0.532	0.595	0.664	0.641	0.685	0.757	0.813	0.870	0.573

The diagonal indicates the square root of the AVE (discriminant validity). The data in the lower triangle correspond to the correlations between the factors. Cr. α, Cronbach’s alpha; CR, composite reliability; AVE, average variance extracted. KNO, knowledge; SKL, skills; CNN, connectedness; CRT, creativity; PAS, passion; TRS, trust; CMM, commitment; VCC, value co-creation.

### Estimation of segment-specific models

A PLS-SEM model was run from a Bootstrapping in SmartPLS 3.0 with the segments obtained. The results are shown in Table 6.

**TABLE 6:** Hypotheses testing.

H	Relationship	Total sample		S1—Detractor customers		S2—Skeptical customers		S3—Neutral customers		S4—Value co-creator customers	
		β	t	β	t	β	t	β	t	β	t
H1	KNO → VCC	0.091*	4.276	0.007	0.052	0.166*	3.676	0.030	0.744	0.065	1.948
H2	SKL → VCC	0.093*	5.315	0.090	0.895	0.161*	4.023	0.064	1.939	0.030	0.976
H3	CNN → VCC	0.145*	6.297	0.275	1.954	0.012	0.240	0.236*	4.555	0.189*	5.027
H4	CRT → VCC	0.090*	3.659	–0.011	0.091	0.003	0.076	0.077	1.584	0.154*	4.083
H5	PAS → VCC	0.149*	5.610	0.153	1.049	0.134**	2.644	0.136**	2.579	0.155*	4.156
H6	TRS → VCC	0.178*	7.664	0.149	1.229	0.277*	5.941	0.122**	2.627	0.088**	2.408
H7	CMM → VCC	0.233*	8.549	0.353**	2.081	0.166*	3.194	0.247*	4.622	0.235*	6.378

*p < 0.005, **p < 0.05.

Table 6 presents the results of the testing of the research hypotheses. When the model is run on the total sample, all the variables corresponding to customer-owned resources and motivations directly impact the intention to co-create brand value. However, when the model is run on the segments obtained through the unobserved heterogeneity, significant differences between types of customers can be seen.

Detractor customers report low intent to brand VCC. In this group, only commitment significantly correlates with the behavior studied. There is a greater desire to co-create value in the skeptical customer segment, and 5 of the 7 proposed hypotheses are positively contrasted. However, they are not customers who co-create brand value.

In segment 3 are the neutral customers, who may be more susceptible to generating behaviors of brand VCC. In this segment, 4 of the 7 proposed hypotheses are positively contrasted, being the first group in which connectivity is presented as a significant antecedent of brand VCC.

Lastly, 5 of the 7 proposed hypotheses are positively contrasted in the segment of customers who co-create brand value. As in the previous group, connectivity is an antecedent of brand VCC, but it is also the only subgroup where creativity significantly affects co-creation intention. Unlike detractor customers, although they score high on brand knowledge and skills, those factors do not positively affect brand VCC. The results are discussed below.

## Discussion

**FIGURE 2:**
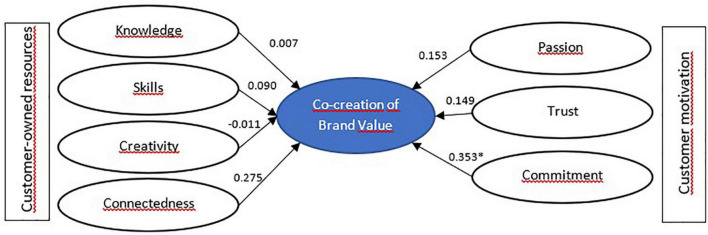
Model estimation for detractor customers—Segment 1. *p < 0.05.

**FIGURE 3:**
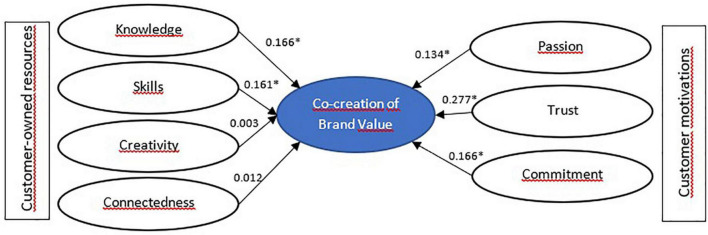
Model estimation for skeptical customers—Segment 2. *p < 0.05.

**FIGURE 4:**
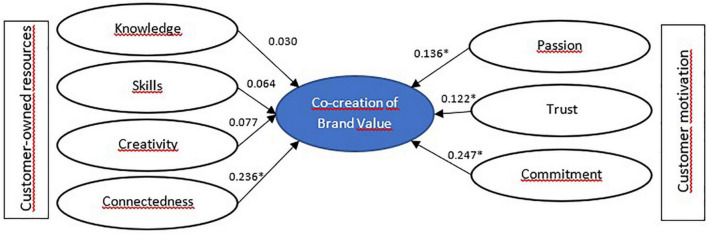
Model estimation for neutral customers—Segment 3. *p < 0.05.

**FIGURE 5:**
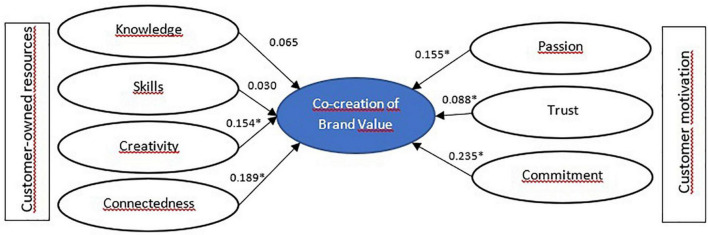
Model estimation for brand value co-creator customers—Segment 4. *p < 0.05.

Results in Table 6 are shown here in Figures 2–5 for segment-specific models.

In the segment of detractor customers, it is evident that only commitment significantly impacts the desired behavior of the variables proposed as antecedents of brand VCC. This first segment comprises customers with little information about the bank and barely trust its skills to perform their job. They are clients who do not show interest in knowing the bank, interacting with other clients, nor have strong positive feelings toward the brand and are not worried about its growth. According to the results of the idiosyncrasy dimensions, it can be deduced that they are passive customers in managing their financial products and do not actively seek preferential rates, good service, or a broad portfolio of financial products.

We noted that customers’ motivations significantly impact the brand VCC behavior for skeptical customers. Also, Knowledge and skills have a strong relationship with the target behavior. However, creativity and connectedness are lacking. We call these consumers skeptical due to their low co-creation of brand value scores despite reporting a high level of knowledge about their bank and high brand skills. They are customers who are not interested in connecting with other bank users and lack creativity.

Regarding their motivations, as seen in Table 4, the values corresponding to passion, trust, and commitment are significantly low, although they have a positive relationship with brand VCC. It can then be understood that the level of trust in the bank is low and, therefore, the commitment to its development and growth is insignificant. On the other hand, this group of customers is more likely to shop around and strongly focuses on the bank’s reputation and the excellent service it can provide. Thus, without being attached to the bank, this customer can be understood as a practical consumer looking for good service and no longer committed to the brand.

Their cultural values present high scores in the categories of universalism, benevolence, and security, values of self-transcendence, and conservation (Schwartz et al., 2017). Although the values related to self-transcendence (universalism and benevolence) are related to the progress of others and the transcendence of selfish interests, the value of conservation (security) is related to the protection of order and harmony in relationships. It is then likely that these clients are motivated by maintaining the *status quo*, hoping to maintain order and protect themselves.

For neutral customers, unlike *skeptical*, the customer-owned resource connectedness has a direct and positive impact on VCC. The motivations for participating are commitment, trust, and passion. The third segment comprises consumers who are halfway in their willingness to co-create. They are an excellent target to reach because their self-report about their bank’s knowledge is high, and their perception of the bank’s ability to fulfill its duties is even higher. Therefore, they need a better and more significant experience with the bank to increase their confidence in improving their positive feelings toward the brand.

As for the idiosyncrasy, they seek an experience with the bank that gives them full transparency in the information that the bank provides, requires a high personalization of the service, and the bank’s reputation is essential. Finally, the most outstanding individual cultural values are benevolence, conformity, and security. Like the previous segment, they are cultural values of the self-transcendent and conservation categories.

For this customer segment, it is observed that it is the only one in which creativity has an impact on the intention to co-create brand value. Therefore, creativity has been pointed out as one of the critical components in brand VCC. Potts et al. (2008) affirm that situated creativity, that is, put to work in specific contexts, such as in the interaction of a client with a company, must be nurtured and promoted through “fluid and permeable” barriers regarding the distribution of capacities of the client and his possibilities of innovation. According to Lewnes and Keller (2019), creativity is vital for any company as it is one of the pillars of innovation. However, creativity does not have to come only from within the company. Companies that facilitate co-creation promote creative processes, making them more competitive.

As in the previous segment, they are customers for whom it is crucial to be connected with other bank users, and they self-report high levels of passion, trust, and commitment. Although these clients self-report excellent knowledge about the bank and sufficient brand skills, these resources do not directly affect the intention to co-create brand value. According to Figure 5, motivational factors are essential.

These are the consumers who report the highest ratings in terms of loyalty. For them, the brand’s reputation, the transparency of information, the portfolio of banking products, good customer service, and crisis management are crucial. While personal cultural values share high scores on benevolence and universalism, like the previous two segments, this segment also reports higher scores on values in the open-to-change category, such as encouragement and self-direction. This category in a previous study showed a negative relationship with the behavioral intention studied (Ahmad et al., 2020).

## Conclusion, implications, and limitations

This study aimed to find the determining factors of brand VCC according to the customers’ idiosyncrasies. To do so, we sampled five different countries with significant samples in each country. We measure the national culture with the Schwartz value scale to ensure the sample’s heterogeneity. Through a quantitative study, we established four types of clients, according to their idiosyncrasies, and national culture, who showed greater or lesser commitment to co-creating value with their banks. The results are very valuable for theory and practice, as discussed below.

When running the model on the total sample, it can be seen that all the hypotheses are positively and significantly contrasted (see Table 6). However, by segmenting the sample according to the unobserved heterogeneity, we could reaffirm what Sarstedt et al. (2022) said; without the study of heterogeneity, it would not have been possible to establish the different customer profiles according to their idiosyncrasies. This highlights how underlying individual differences in clients can challenge behavioral theories. Therefore, this work demonstrates the need to carry out studies on the unobserved heterogeneity in behavioral research, especially in large samples like the one in the present study.

Additionally, in the discussion about national culture and its impact on consumer attitudes and behaviors, according to Ahmad et al. (2020), some consumers’ attitudes and behaviors (such as eco-friendly consumption) may be positively related to self-transcendence and conservation values and negatively associated with self-enhancement and openness-to-change values. However, this study shows an inverse relationship between these categories and the behavior studied. Therefore, more studies about cultural values and their impact on consumer behavior across markets and economic sectors may be necessary.

The study results provide relevant information for the industry since no similar study is available. Understanding the consumer from their most personal motivations for interaction and brand VCC is one of the significant contributions of this research. According to the results, commitment is essential in all customer profiles to co-create value with banks. However, for co-creation to finally occur, it is necessary to have a passion for the brand, accompanied by the consumer’s resources such as creativity and connectivity. In addition, the knowledge about the brand and the skills perceived by the consumer about the company are not enough to carry out co-creation behaviors in the banking sector.

The research was developed in the banking sector as it is one with the most significant difficulties in generating customer loyalty. However, it may differ in other contexts, where customers’ negative emotions toward brands are not as strong. For example, there may be significant differences in studies with clients of luxury brands, where brand building is done through identifying personality, the extended-self, the feeling of exclusivity, and, sometimes, aspirational consumption. In such contexts, customers are more likely to co-create brand value since they identify with the brand, their passion will be much more significant, and they will be committed to making the brand grow and gaining recognition. In the luxury sector, studies have shown that the experience with the brand is hedonic and that the greater the pleasurable experience, the greater the intention to co-create with the brand (Chapman and Dilmperi, 2022). In the case of the banking sector, the possibility of creating pleasurable experiences is challenging. Hence the importance of including idiosyncrasies understood as the set of customers’ beliefs and attitudes due to their prior experiences. Banks have traditionally presented themselves as cold, impersonal institutions that do not care about the client’s wellbeing and have the last word in people’s financial lives. This is why if banks want to build better relationships with their customers and win their loyalty, they must rethink their ways, approaches, and, especially, their customers’ experience.

### Limitations and future research

Although we used a broad sample, the model was adapted from Merz et al. (2018); considering the dimensions of customer-owned resources and motivations to co-create brand value, other factors that could also drive the behavior studied have not been taken into account.

Although the analysis of the unobserved heterogeneity gives essential information about behavior by segments, in this investigation, we only used the idiosyncrasy to generate profiles. Sociodemographic elements that could be part of another study were not considered.

On the other hand, we do not have evidence about the banks’ initiatives to motivate co-creation processes more than providing information to the client. Variables directly studied in banks, such as programs for the co-creation of value with customers, communication of these programs, the inclusion of customers in production, and active participation of customers in the processes, among others, could give a broader look at the adoption of co-creation processes in the banking sector.

The study of brand VCC also encourages the study of relevant issues such as sustainability, digital transformation, and green practices. Furthermore, by including the client in co-creation processes and improving knowledge management practices, positive impacts can be had on companies’ innovation, market, and financial performance (Castagna et al., 2020). Furthermore, studies focused on the benefits of co-creation will allow companies to understand and accept the inclusion of external agents to the organization in their processes (Abbate et al., 2019), even when an immediate investment is required since the benefits in the medium and long term are profuse.

## Data availability statement

The datasets presented in this study can be found in online repositories. The names of the repository/repositories and accession number(s) can be found below: doi: 10.17632/wmm4sprjhr.1.

## Ethics statement

The studies involving human participants were reviewed and approved by CESA Ethics Committee. The patients/participants provided their written informed consent to participate in this study.

## Author contributions

NP-G and ML-O provided the conceptualization, data collection, and initial analysis. JJ-R worked on the results and methodology. NP-G provided writing. ML-O contributed to review and editing. All authors contributed to this study, read and agreed to the submitted version of the manuscript.

## References

[B1] Aaker D. A. (1996). Measuring brand equity across products and markets. *Calif. Manage. Rev.* 38 102–120. 10.2307/41165845

[B2] Abbate T. Codini A. P. Aquilani B. (2019). Knowledge co-creation in open innovation digital platforms: Processes, tools and services. *J. Bus. Indus. Market.* 34 1434–1447. 10.1108/JBIM-09-2018-0276

[B3] Ahmad W. Kim W. G. Anwer Z. Zhuang W. (2020). Schwartz personal values, theory of planned behavior and environmental consciousness: How tourists’ visiting intentions towards eco-friendly destinations are shaped? *J. Bus. Res.* 110 228–236. 10.1016/j.jbusres.2020.01.040

[B4] Alam M. M. D. Karim R. A. Habiba W. (2021). The relationship between CRM and customer loyalty: The moderating role of customer trust. *Int. J. Bank Market.* 39 1248–1272. 10.1108/IJBM-12-2020-0607

[B5] Al-Kumaim N. H. Alhazmi A. K. Ramayah T. Shabbir M. S. Gazem N. A. (2021). Sustaining continuous engagement in value co-creation among individuals in universities using online platforms: Role of knowledge self-efficacy, commitment and perceived benefits. *Front. Psychol.* 12:637808. 10.3389/fpsyg.2021.637808 33643168 PMC7907507

[B6] Brandtzæg P. B. (2010). Towards a unified Media-User Typology (MUT): A meta-analysis and review of the research literature on media-user typologies. *Comp. Hum. Behav.* 26 940–956.

[B7] Brewer P. Venaik S. (2012). On the misuse of national culture dimensions. *Int. Market. Rev.* 29 673–683. 10.1108/02651331211277991

[B8] Brodbeck F. C. Chhokar J. S. House R. J. (2013). *Culture and leadership across the world.* England, UK: Routledge.

[B9] Bruce H. L. Wilson H. N. Macdonald E. K. Clarke B. (2019). Resource integration, value creation and value destruction in collective consumption contexts. *J. Bus. Res.* 103 173–185. 10.1016/j.jbusres.2019.05.007

[B10] Burton D. (2008). *Cross-cultural marketing: Theory, practice and relevance.* England, UK: Routledge.

[B11] Cambra-Fierro J. Pérez L. Grott E. (2017). Towards a co-creation framework in the retail banking services industry: Do demographics influence? *J. Retail. Consume. Serv.* 34 219–228. 10.1016/j.jretconser.2016.10.007

[B12] Carolina? (2019). Dimensions of national culture–cross-cultural theories. *Stud. Bus. Econ.* 14 220–231.

[B13] Casper Ferm L.-E. Thaichon P. (2021). Value co-creation and social media: Investigating antecedents and influencing factors in the U.S. retail banking industry. *J. Retail. Consume. Serv.* 61:102548. 10.1016/j.jretconser.2021.102548

[B14] Castagna F. Centobelli P. Cerchione R. Esposito E. Oropallo E. Passaro R. (2020). Customer knowledge management in SMEs facing digital transformation. *Sustainability* 12:3899.

[B15] Chapman A. Dilmperi A. (2022). Luxury brand value co-creation with online brand communities in the service encounter. *J. Bus. Res.* 144 902–921. 10.1016/j.jbusres.2022.01.068

[B16] Engelen A. Brettel M. (2011). Assessing cross-cultural marketing theory and research: Reply to Craig and Douglas’ commentary. *J. Bus. Res.* 64 782–784.

[B17] Fink L. (2022). *Larry Fink’s annual 2022 letter to CEOs.* New York, NY: BlackRock.

[B18] FinTech (n.d.). *Worldwide | Statista Market Forecast Statista.* Available online at: https://www-statista-com.cvirtual.cesa.edu.co/outlook/dmo/fintech/worldwide (accessed on Jan 11, 2022).

[B19] Fourie I. (2012). Understanding and exploiting idiosyncrasy in the use of ICT devices such as tablets: Setting the background. *Libr. Hi Tech.* 30 359–366. 10.1108/07378831211240012

[B20] Fuentes-Blasco M. Moliner-Velázquez B. Gil-Saura I. (2014). Effect of customer heterogeneity on the relationship satisfaction–loyalty. *Revista Española de Investigación de Market.* ESIC 18 78–92.

[B21] Gabriel A. S. Daniels M. A. Diefendorff J. M. Greguras G. J. (2015). Emotional Labor Actors: A Latent Profile Analysis of Emotional Labor Strategies. *J. Appl. Psychol.* 100 863–879. 25068812 10.1037/a0037408

[B22] Gorgoglione M. Panniello U. (2018). Beyond customer experience models: Identifying idiosyncratic perceptions. *Int. J. Bank Market.* 36 1311–1328. 10.1108/IJBM-06-2017-0124

[B23] Guo C. J. Warkentin M. Luo X. R. Gurung A. Shim J. P. (2020). An imposed etic approach with Schwartz polar dimensions to explore cross-cultural use of social network services. *Inform. Manage.* 57:103261.

[B24] Haffar M. Enongene L. Gbadamosi G. Hamdan M. (2016). The influence of national culture on consumer buying behaviour: An exploratory study of Nigerian and British consumers. *WASET Int. J. Soc. Behav. Educ. Econ. Manage. Engin.* 10 3033–3037.

[B25] Hart S. Young R. A. (2014). *Contemporary Latin American cultural studies.* England, UK: Routledge.

[B26] Hassan H. E. Wood V. R. (2020). Does country culture influence consumers’ perceptions toward mobile banking? A comparison between Egypt and the United States. *Telem. Inform.* 46:101312. 10.1016/j.tele.2019.101312

[B27] Hofstede G. (1994). Management scientists are human. *Manage. Sci.* 40 4–13.

[B28] Hofstede G. (2009). *Geert hofstede cultural dimensions*. Mexico: Taylor training.

[B29] Hofstede G. (2011). Dimensionalizing cultures: The Hofstede model in context. *Online Read. Psychol. Cult.* 2:8.

[B30] Hofstede G. Bond M. H. (1988). The Confucius connection: From cultural roots to economic growth. *Organ. Dynam.* 16 5–21.

[B31] Hollander E. P. (1958). Conformity, status, and idiosyncrasy credit. *Psychol. Rev.* 65 117–127. 10.1037/h0042501 13542706

[B32] Hroch M. (1998). *From national movement to the fully-formed nation.* Sparkford: Haynes M.

[B33] Karpen I. O. Bove L. L. Lukas B. A. (2012). Linking service-dominant logic and strategic business practice: A conceptual model of a service-dominant orientation. *J. Serv. Res.* 15 21–38.

[B34] Kleinaltenkamp M. Brodie R. J. Frow P. Hughes T. Peters L. D. Woratschek H. (2012). Resource integration. *Market. Theory* 12 201–205.

[B35] Kozinets R. V. Hemetsberger A. Schau H. J. (2008). The wisdom of consumer crowds: Collective innovation in the age of networked marketing - Robert V. Kozinets, Andrea Hemetsberger, Hope Jensen Schau, 2008. *J. Macromarket.* 28 339–354.

[B36] Kumar V. Pansari A. (2016). National culture, economy, and customer lifetime value: Assessing the relative impact of the drivers of customer lifetime value for a global retailer. *J. Int. Market.* 24 1–21.

[B37] Latin America and Caribbean: GDP by country 2020 (n.d.). *Statista.* Available online at: https://www.statista.com/statistics/802640/gross-domestic-product-gdp-latin-america-caribbean-country/ (accessed on Jan 11, 2022).

[B38] Lee I. Shin Y. J. (2018). Fintech: Ecosystem, business models, investment decisions, and challenges. *Bus. Horiz.* 61 35–46. 10.1016/j.bushor.2017.09.003

[B39] Lewnes A. Keller K. L. (2019). 10 Principles of Modern Marketing - ProQuest. *MIT Sloan Manage. Rev.* 60 1–10.

[B40] Mazzucato M. (2022). *Larry Fink’s capitalist shell game | by Mariana Mazzucato.* New York, NY: Project Syndicate.

[B41] Merhabi M. A. Petridis P. Khusainova R. (2021). Gamification for brand value co-creation: A systematic literature review. *Information* 12 345–363. 10.3390/info1209034

[B42] Merz M. A. Zarantonello L. Grappi S. (2018). How valuable are your customers in the brand value co-creation process? The development of a Customer Co-Creation Value (CCCV) scale. *J. Bus. Res.* 82 79–89. 10.1016/j.jbusres.2017.08.018

[B43] Moghadamzadeh A. Ebrahimi P. Radfard S. Salamzadeh A. Khajeheian D. (2020). Investigating the role of customer co-creation behavior on social media platforms in rendering innovative services. *Sustainability* 12:6926.

[B44] Monavvarifard F. Baradaran M. Khosravipour B. (2019). Increasing the sustainability level in agriculture and Natural Resources Universities of Iran through students’ engagement in the value Co-creation process. *J. Clean. Product.* 234 353–365.

[B45] Moschieri C. Campa J. M. (2014). New trends in mergers and acquisitions: Idiosyncrasies of the European market. *J. Bus. Res.* 67 1478–1485. 10.1016/j.jbusres.2013.07.018

[B46] Nysveen H. Pedersen P. E. (2014). Influences of cocreation on brand experience. *Int. J. Mark. Res.* 56 807–832.

[B47] Payne A. F. Storbacka K. Frow P. (2008). Managing the co-creation of value. *J. Acad. Mark. Sci.* 36 83–96. 10.1007/s11747-007-0070-0

[B48] Payne E. M. Peltier J. W. Barger V. A. (2018). Mobile banking and AI-enabled mobile banking: The differential effects of technological and non-technological factors on digital natives perceptions and behavior. *J. Res. Interact. Mark.* 12, 328–346

[B49] Pinho N. Beirão G. Patrício L. Fisk P. R. (2014). Understanding value co-creation in complex services with many actors. *J. Serv. Manage.* 25 470–493. 10.1108/JOSM-02-2014-0055

[B50] Pohlmann A. Kaartemo V. (2017). Research trajectories of Service-Dominant Logic: Emergent themes of a unifying paradigm in business and management. *Indus. Market. Manage.* 63 53–68.

[B51] Potts J. Hartley J. Banks J. Burgess J. Cobcroft R. Cunningham S. (2008). Consumer Co-creation and Situated Creativity. *Indus. Innov.* 15 459–474. 10.1080/13662710802373783

[B52] Prahalad C. K. Ramaswamy V. (2004). Co-creation experiences: The next practice in value creation. *J. Interact. Market.* 18 5–14. 10.1002/dir.20015

[B53] Rehman V. (2017). Looking through the glass of Indian culture: Consumer behaviour in modern and postmodern era. *Glob. Bus. Rev.* 18 S19–S37.

[B54] Robinson T. D. (2019). *Time and culture in consumer behaviour: Framing the future.* England, UK: Routledge.

[B55] Sarstedt M. Radomir L. Moisescu O. I. Ringle C. M. (2022). Latent class analysis in PLS-SEM: A review and recommendations for future applications. *J. Bus. Res.* 138 398–407. 10.1016/j.jbusres.2021.08.051

[B56] Schwartz S. H. (1994). “Beyond individualism/collectivism: New cultural dimensions of values,” in *Individualism and collectivism: Theory, method, and applications Cross-cultural research and methodology series, 18*, eds Kim U. Triandis H. C. Kagitcibasi C. Choi S. C. Yoon G., (Thousand Oaks, CA: Sage Publications, Inc), 85–119.

[B57] Schwartz S. H. (1999). A theory of cultural values and some implications for work. *Appl. Psychol.* 48 23–47. 10.1111/j.1464-0597.1999.tb00047.x

[B58] Schwartz S. H. (2012). An Overview of the Schwartz Theory of Basic Values. *Online Read. Psychol. Cult.* 2 2307–0919. 10.9707/2307-0919.1116

[B59] Schwartz S. H. Cieciuch J. Vecchione M. Torres C. Dirillen-Gumus O. Butenko T. (2017). Value tradeoffs propel and inhibit behavior: Validating the 19 refined values in four countries. *Eur. J. Soc. Psychol.* 47 241–258. 10.1002/ejsp.2228

[B60] Steers R. M. Meyer A. D. Sanchez-Runde C. J. (2008). National culture and the adoption of new technologies. *J. World Bus.* 43 255–260. 10.1016/j.jwb.2008.03.007

[B61] Tung R. L. Verbeke A. (2010). Beyond Hofstede and GLOBE: Improving the quality of cross-cultural research. *J. Int. Bus. Stud.* 41 1259–1274. 10.1057/jibs.2010.41

[B62] Vargo S. L. Lusch R. F. (2004). Evolving to a New Dominant Logic for Marketing. *J. Market.* 68 1–17. 10.1509/jmkg.68.1.1.24036 11670861

[B63] Vargo S. L. Lusch R. F. (2016). Institutions and axioms: An extension and update of service-dominant logic | SpringerLink. *J. Acad. Market. Sci.* 44 5–23.

[B64] Vargo S. L. Lusch R. F. (2017). Service-dominant logic 2025. *Int. J. Res. Market.* 34 46–67. 10.1016/j.ijresmar.2016.11.001

[B65] Vargo S. L. Maglio P. P. Akaka M. A. (2008). On value and value co-creation: A service systems and service logic perspective. *Eur. Manage. J.* 26 145–152.

[B66] Williams R. (2014). *Keywords: A vocabulary of culture and society.* Oxford, UK: Oxford University Press.

